# Comparison of effectiveness of cefovecin, doxycycline, and amoxicillin for the treatment of experimentally induced early Lyme borreliosis in dogs

**DOI:** 10.1186/s12917-015-0475-9

**Published:** 2015-07-25

**Authors:** Bettina Wagner, John Johnson, David Garcia-Tapia, Nicole Honsberger, Vickie King, Catherine Strietzel, John M. Hardham, Thomas J. Heinz, Richard T. Marconi, Patrick F. M. Meeus

**Affiliations:** Department of Population Medicine and Diagnostic Sciences, College of Veterinary Medicine, Cornell University, Ithaca, NY USA; Veterinary Medicine Research and Development, Zoetis, 333 Portage Street, Kalamazoo, MI 49007 USA; Department of Microbiology and Immunology, Virginia Commonwealth University Medical Center, Richmond, PO Box 980678, VA 23298 USA

**Keywords:** Lyme Disease, *Borrelia burgdorferi*, Doxycycline, Amoxicillin, Cefovecin

## Abstract

**Background:**

While Koch’s postulates have been fulfilled for Lyme disease; causing transient fever, anorexia and arthritis in young dogs; treatment of sero-positive dogs, especially asymptomatic animals, remains a topic of debate. To complicate this matter the currently recommended antibiotic treatments of Lyme Disease in dogs caused by *Borrelia burgdorferi* require daily oral administrations for 31 days or longer, which makes non-compliance a concern. Additionally, there is no approved veterinary antimicrobial for the treatment of Lyme Disease in dogs in the USA and few recommended treatments have been robustly tested.

*In vitro* testing of cefovecin, a novel extended-spectrum cephalosporin, demonstrated inhibition of spirochete growth. A small pilot study in dogs indicated that two cefovecin injections two weeks apart would be as efficacious against *B. burgdorferi* sensu stricto as the recommended treatments using doxycycline or amoxicillin daily for 31 days.

This hypothesis was tested in 17–18 week old Beagle dogs, experimentally infected with *B. burgdorferi* sensu stricto, using wild caught ticks, 75 days prior to antimicrobial administration.

**Results:**

Clinical observations for lameness were performed daily but were inconclusive as this characteristic sign of Lyme Disease rarely develops in the standard laboratory models of experimentally induced infection. However, each antibiotic tested was efficacious against *B. burgdorferi* as measured by a rapid elimination of spirochetes from the skin and reduced levels of circulating antibodies to *B. burgdorferi*. In addition, significantly less cefovecin treated animals had Lyme Disease associated histopathological changes compared to untreated dogs.

**Conclusions:**

Convenia was efficacious against *B. burgdorferi* sensu stricto infection in dogs as determined by serological testing, PCR and histopathology results. Convenia provides an additional and effective treatment option for Lyme Disease in dogs.

## Background

In North America, Lyme Disease (LD) in dogs is caused by *Borrelia burgdorferi* (*Bb*) sensu stricto a tick-borne spirochete. A diagnosis of LD is made based on an assessment of tick exposure risk, clinical signs consistent with disease, serological testing, differential diagnosis and response to antimicrobial therapy [1]. Clinical signs of LD are generally non-descript in dogs, but may include fever, arthritis, anorexia, lymphadenopathy and glomerulonephritis [2, 3]. However, the majority of dogs sero-positive to *Bb* do not show clinical signs of the disease [1]. There are no approved antimicrobials for the treatment of LD in dogs and because it is difficult to induce clinical disease in dogs by experimental infection, the optimal use of available antibiotics and duration of treatment are unknown [1]. Not only do opinions vary on how to treat dogs, but also on whether or not to treat sero-positive, but asymptomatic dogs. Additionally, all currently recommended treatments require frequent and prolonged antibiotic administration which makes owner non-compliance with the full dosing regimen a concern [4].

The objective of this study was to compare the outcome of treatment of beagles experimentally infected with *Bb* with cefovecin, a novel long-acting cephalosporin [5, 6], administered as two subcutaneous injections 14 days apart to the recommended daily administration of doxycycline or amoxicillin for 31 days [1]. In this study we used an “early” LD model that is based on histopathological changes in joint synovial tissues [7–9]. This model consistently and reliably produces joint lesions which precede the development of LD induce lameness. The United States Department of Agriculture (USDA) Center for Veterinary Biologics (CVB) has accepted this early LD model as the primary variable in support of label claims against subclinical arthritis associated with LD for vaccine registration purposes (CVB. Demonstrating efficacy against canine Lyme disease. Veterinary Services Memorandum Draft No. 316, 2007). Clinical signs, serological responses and the presence or absence of *Borrelia* spirochete DNA in skin biopsies were also measured to provide broad and clinically relevant measurements of response to treatment.

## Results

### Cefovecin *in vitro* growth inhibition of spirochetes

**Fig. 1:**
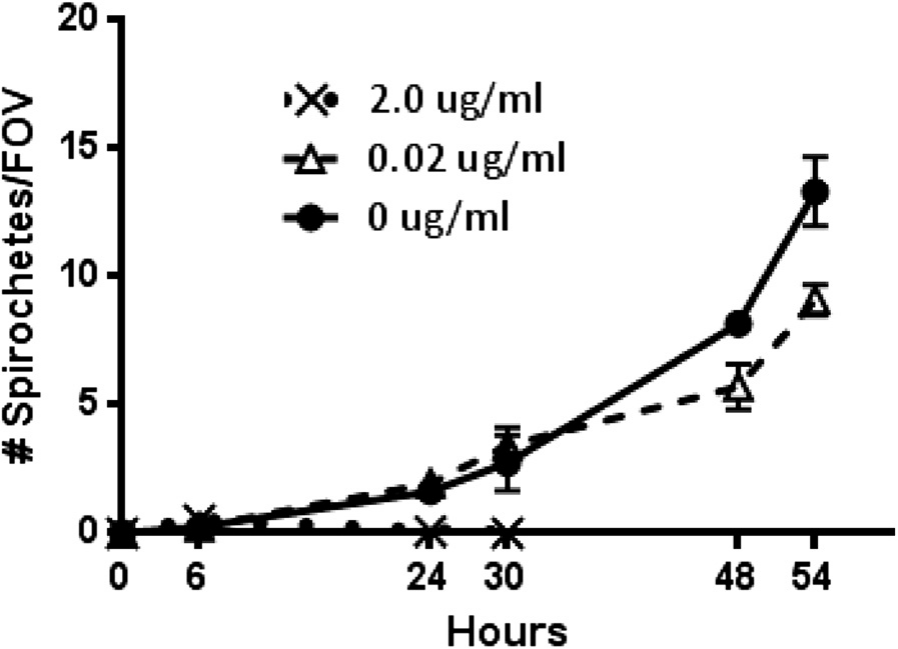
Cefovecin *in vitro* growth inhibition of *Bb* viability. *Bb* viability in the presence of 0, 0.2 or 2 ug/ml cefovecin was determined at 0, 6, 24, 30, 48, and 54 h by counting motile spirochetes in ten random field-of-views (FOV) using phase contrast microscopy. Each dose was assessed in triplicate and the mean calculated; error bars represent the standard error of the mean (SEM), when error bars are not shown the SEM is smaller than the symbols

Cefovecin at a high concentration of 2 ug/ml eliminates *Borrelia burgdorferi* strain B31 by 24–30 h in BSK-H culture at 34C, and 0.02 ug/ml of cefovecin retards spirochete growth by at least 30 % in 54 h (Fig. 1). Data from this limited initial probe study bracket the MIC of cefovecin against a high pass *B. burgdorferi* lab strain.

### Detection of spirochete DNA in skin biopsies

Polymerase chain reaction (PCR) amplification of the *Borrelia flaB* gene confirmed the successful infection of dogs with spirochetes from the wild caught ticks by day 48, with 12 out of 32 dogs having detectable *Borrelia* DNA in their skin biopsies. This ratio continued to increase in untreated dogs through day 118, with 6 out of 8 dogs becoming positive, but declined thereafter to become negative for all untreated dogs by day 180. None of the treated animals remained PCR positive after treatment with doxycycline, amoxicillin or cefovecin started on day 75. This difference in frequency was significant between the untreated controls and the treated groups on day 118 only. No significant differences were observed between the PCR results of any of the antibiotic treatment groups.

### Clinical signs

For three weeks after tick challenge body temperatures were measured daily in all dogs. Five to eight dogs in each group had pyrexia (>39.5 °C) at least one time during that period, but all dogs remained bright and alert. Only one dog from the untreated group was lame on day 196 and one dog from the doxycycline-treated group was lame on day 264.

### Serological responses to infection and treatment

**Fig. 2:**
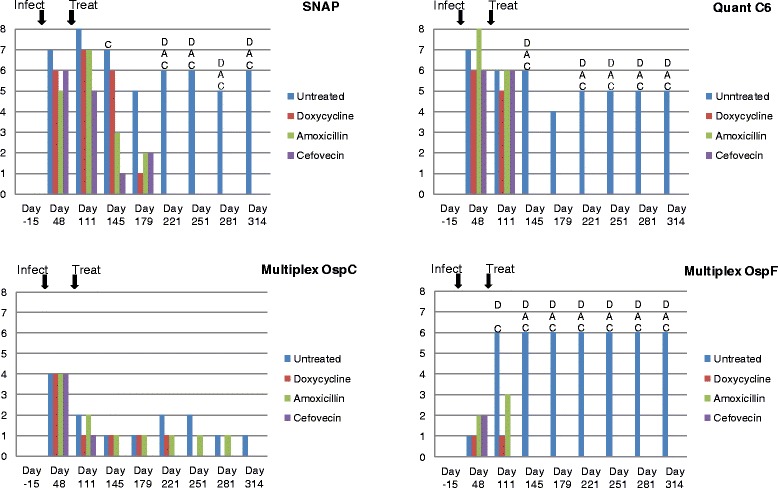
Number of *Bb* sero-positive treated and untreated dogs using different antigens and diagnostic platforms. The number of sero-positive dogs in each of 4 treatment groups (*n* = 8) using 4 routinely used commercial assays at different times after infections and treatments. Statistical analysis was only possible after day 48, because of housing restrictions. Statistical difference of a treatment group from untreated animals is designated by D (Doxycycline), A (Amoxicillin) and C (Cefovecin)

Prior to tick infestation (study day −15) all dogs were serologically negative for *Bb*-specific antibodies using several independent assays (Fig. 2). By day 48, i.e., prior to treatment, 5–7 animals in each group had become SNAP positive (Fig. 2). The dogs continued to sero-convert even after treatment with the highest number of SNAP positive dogs observed on day 111, about 15 weeks after the start of tick infestation and 5 weeks after the start of antimicrobial treatments. Subsequently the numbers of SNAP sero-positive dogs in the treated groups declined to zero by day 221 or around 21 weeks after the start of the treatment. In contrast, in the untreated control group only 2–3 dogs became sero-negative on any given sampling day for the remainder of the study. The difference in the number of SNAP positive dogs between untreated and treated animals was significant on day 145 for the cefovecin group only. By day 221, however, all antibiotic treated groups had significantly less sero-positive dogs than the untreated control group. Also, on Day 145 the cefovecin group had significantly fewer dogs positive than the doxycycline group. At no other time was a significant difference observed between any of the antibiotic treatments used in the number of SNAP positive dogs.

Antibody values using the Quant C_6_ assays followed a similar pattern as the SNAP test and many animals passed the sero-positive cut-off limits by day 48 (Fig. 2). In contrast to the SNAP test, however, all treated animals became Quant C_6_ negative by day 145, irrespective of the antibiotic used. This difference in frequency was significant on all subsequent sampling days, except on day 179 when only 4 untreated animals were sero-positive.

Antibody values in the Lyme Multiplex assay reflect the different expression stages of the surface antigens used. OspA, an antigen expressed in the tick vector, only stimulated an early and transient serological response below the positive cut-off level and differences were not detected in any of the samples (data not shown). OspC, expressed during transmission of the pathogen from the tick vector to the mammalian host, generated a serological response early in the infection, but only in half of the animals. The antibodies to OspC subsequently declined in all animals, including those in the untreated control group (Fig. 2). No significant differences were observed between any of the groups at any time point. By contrast, antibodies to OspF appeared later in the infection, peaking around day 111 in the untreated controls, and only declined upon treatment (Fig. 2). By day 111, all animals in the cefovecin treated group had already become negative for OspF antibodies, which was significantly lower than the untreated control group with only 2 negative animals. At the next sampling point all amoxicillin and doxycycline treated animals also became negative for OspF antibodies, while the number of sero-negative untreated controls remained significantly lower for the rest of the trial with only 2 out of 8 animals.

### Histopathological evaluation of skin and joint synovial tissues

**Fig. 3:**
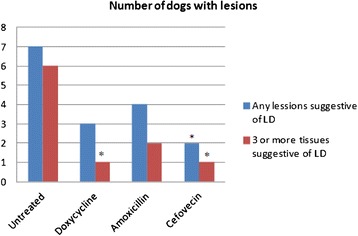
Number of dogs with lesions associated with Lyme Disease. The number dogs with any lesions associated with LD in tissue samples from the joint capsule or synovium from left and right shoulder, elbow, carpus, stifle, and tarsus of each dog or dogs with lesions in more than 3 tissues was determined in each treatment group on day 315. *Statistically different treated dogs from untreated animals

**Fig. 4:**
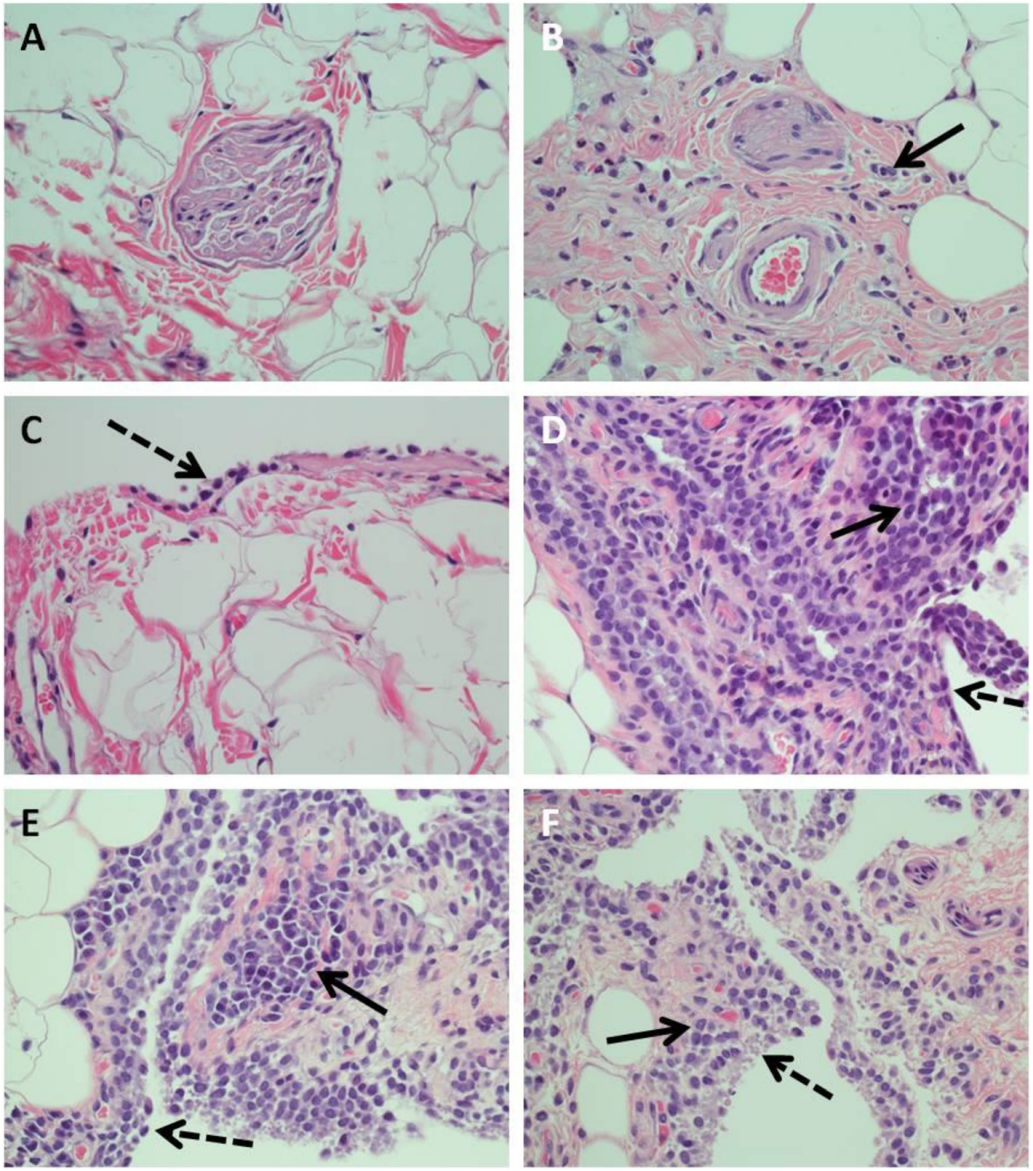
Characteristic histopathological lesions in subdermis and synovial joint capsules of treated and non-treated animals challenged with *Bb.* Non-affected skin-subdermis and synovial capsule are depicted in panels (**a**) and (**c**) respectively. Panel **b** shows a section of subdermis with minimal inflammation in the perivascular and perineural tissue (pointed by the solid arrow). Note the absence of this infiltrate in (**a**). Panels **d** to **f** show inflammatory changes observed in joint capsule from three different dogs. The solid arrows point at areas with mononuclear inflammation. Note the nodular appearance of the inflammation composed mainly by lymphocytes and plasma cells in panel (**e**). The dashed arrows point at the synovial layer, which is composed by a single-cell layer in the tissue in panel (**c**). Note the absence of inflammatory infiltrate in this tissue. All pictures were taken at a 400 x total magnification

The number of dogs, per treatment group, identified with lesions consistent with LD is shown in Fig. 3. Histological sections of healthy skin and joint tissue (panels a and c), as well as lesions typical associated with LD (panels b, d, e, f) found in untreated dogs are show in Fig. 4. These lesions are characterized by inflammation of the perivascular and perineural tissue in the skin and by nodular inflammation composed mainly by lymphocytes and plasma cells in the joint capsules. When considering any lesion as possibly associated with LD, 7 out of 8 control dogs were considered affected, while the doxycycline, amoxicillin and cefovecin treated groups only had 3, 4 and 2 affected animals, respectively (Fig. 3). This reduction in affected animals, when compared to the untreated controls, was only significant in the cefovecin treated group. When only taking into account those animals that had at least 3 tissues affected with LD associated lesions, as a means to remove normal background changes in the joints, both doxycycline and the cefovecin treated groups achieved significance when compared to the untreated controls (Fig. 3). The amoxicillin treated group never achieved a significant reduction in the number of affected animals when compared to the controls, nor was any significant difference observed between any of the treatment groups.

## Discussion

The purpose of this study was to evaluate the clinical effectiveness of cefovecin against early LD in an experimental model using wild caught ticks. While the presence of other pathogens cannot be excluded in these wild caught ticks, the presence of *B. burgdorferi* sensu stricto, general considered as the causative agent of LD, was confirmed in both ticks and dogs. Screening MIC data for a number of leptospira species and a small pilot study conducted at Zoetis suggested that cefovecin at the FDA registered dose (8 mg/kg) and interval (2 doses at 14 days interval) would be effective. To bridge the current results to previous studies [10, 11] and to compare cefovecin to the antibiotics currently considered the standard of care [1] we also included doxycycline and amoxicillin treatments in this study.

Given that predictably eliciting overt clinical disease, such as lameness, in dogs in an experimental model has eluded researchers to date [7, 8, 12], we relied in this study on evaluating the presence or absence of the pathogens using PCR amplification of *Borrelia* DNA in skin biopsies, serological responses and histopathological changes in skin and joint tissue to test the effectiveness of the antibiotics.

As observed previously, very few dogs in this study developed clinical signs associated with LD. Only two dogs became transiently lame: one dog from the untreated group on day 196 post *Bb* exposure and one dog 189 days after treatment with doxycycline. The lack of overt clinical signs occurred despite successfully infecting all 32 dogs as evidenced by the presence of *Bb*-specific antibodies in at least one serological test (SNAP, Quant C_6_ or Multiplex) or a positive PCR from skin biopsies. Review of the individual histopathology results in the two dogs with lameness revealed that only 2 of 10 joints examined, the right stifle and the left tarsus, had any lesions compatible with those described for LD in the doxycycline treated dog, with all lesions characterized as mild. However, the untreated control dog had lesions compatible with those described for LD in 9 out of 10 joints examined, with lesions in 4 of these joints; the right carpus, the left shoulder, and the left and right tarsus; characterized as moderate to marked in severity.

Similar serological responses to infection and treatment were observed across all diagnostic platforms and antigens used. Differences observed are associated with the change in expression of antigens by the *Borrelia* spirochetes as they transition from the tick vector to the mammalian host and subsequently adapt to the host and its mounting immune response [13]. As such antibodies to OspC, a surface antigen associated with the initial invasion of the dogs, appear early in the infection only and antibody values drop typically by 6–8 weeks post infection [13]. Treatment in this study started on day 75 post tick exposure. As expected, antibodies to OspC subsequently increased after infection and had started to drop in all groups, including the untreated control group by the next sampling time point on day 111 post infection. OspC is therefore an excellent marker for early or recent infection, but on its own is not suited to measure the response to treatment that is applied as late into infection as in this study. By contrast, both the C_6_ and OspF antigens are expressed during later infection and appear to be good markers to measure treatment success [13–15]. Antibodies to both markers remain relatively high and above the sero-positive cut-off limits in many untreated control animals for at least 300 days after infection. In contrast, the antibiotic treated groups no longer had substantial antibodies circulating to these antigens within 70 days of antibiotic treatment. While the pathogenesis and the role of circulating antibodies of LD associated protein-losing nephropathy remains unclear [1, 16–19] the significant reduction in circulating antibodies to Bb antigens might provide clinical benefits [19, 20] and is considered an indicator of a reduced bacterial burden.

Inflammation in the synovium or in joint capsules along with hypertrophy/hyperplasia of the synovial layer has been observed in dogs diagnosed with experimental LD and these lesions are considered to be precursors to the lameness typically associated with the infection [8, 12]. In this study the cefovecin-treated group had the fewest number of dogs with lesions and this number was significantly lower than the untreated controls. Given that both doxycycline and amoxicillin are generally considered as efficacious against *Borrelia* [1] and given the relatively small number of dogs per group, it is not surprising that no significant difference was observed between any of the antibiotic treatment groups. While it is encouraging that even 225 days after the last cefovecin treatment significantly less treated animals had joint lesions associated with LD, this study did not assess the longer term benefits of treatment. In previous studies *Borrelia* antibodies rose again 150–180 days after a 30 day treatment with amoxicillin or doxycycline [21], suggesting that the infection had not been eliminated from the animals and that the possibility of clinical and pathological relapse remained. While we did not observe such rise in this study, we cannot exclude that longer observations or immune-suppression with corticosteroids [11] might be necessary to determine whether or not the pathogens were eliminated from the animals or not.

## Conclusions

Cefovecin administered as two subcutaneous injections at 8 mg/kg body weight 14 days apart provided a significant and sustained reduction in the numbers of dogs with joint lesions and circulating antibodies associated with LD infection. This outcome was comparable to that observed with 31 days of daily oral doxycycline or amoxicillin administration.

## Methods

### Ethics

The study and the experimental design were approved by the Zoetis Institutional Animal Care and Use Committee (IACUC).

### *In vitro* testing of Cefovecin

*Borrelia burgdorferi* strain B31 (ATCC 35210) spirochetes were grown at 34 °C under microaerophilic conditions in modified Barbour-Stoenner-Kelly (BSK-H) Medium Complete (Sigma, St. Louis, MO, USA). Because minimum inhibitory concentrations of most 3^rd^ generation cephalosporins against a variety of *Borrelia* species fall within ≤0.015 and 0.125 mcg/ml [22–25], a low and high concentration approximately covering this range were selected for testing. Cefovecin 100X stocks were prepared in water and sterile-filtered (0.22 micron). The same volume of each stock solution, 40 ul, was added to 4 ml of spirochetes in media for final cefovecin dilutions of 0, 0.02 and 2.0 mcg/ml. Triplicates were prepared for each of the three treatments and monitored for growth over a three day period. Time points for sampling were at 0, 6, 24, 30, 48, and 54 h. Spirochete viability in each sample was based on total motile spirochetes by counting ten random field-of-views (FOV) via phase contrast microscopy [26].

### Animals

Thirty-two healthy, purpose-bred, 17–18 week old Beagle dogs were used in this study. The animals received all routine vendor specific and Zoetis site specific vaccinations; but care was taken to exclude vaccines against LD or leptospirosis. All dogs were also tested, 15 days prior to experimental tick exposure, for antibodies to *Bb* using the SNAP assay and found to be negative.

### Study groups, tick exposure and antimicrobial treatment

This was a prospective, masked, randomized study in dogs experimentally infected with *Bb*. Dogs were acclimated to the Zoetis study facility for at least seven days prior to the start of study activities. Prior to day 0 dogs were allocated to treatments and runs according to a randomization plan produced by a Zoetis Biometrician using a split-plot design with a completely random design in the whole plot (sex) and a randomized complete block in the split-plot (treatment) in multiple rooms. For the duration of the tick infestation period the dogs were housed individually to avoid removal of ticks. On study day 55, after the infestation period and before the treatment period, the dogs were moved to dual housing based on sex (male-male and female-female) to allow for more social interaction for the duration of the study as recommended by Zoetis standard operating procedures for long term studies.

In April of 2010, study day 0, all 32 dogs were individually exposed to 20 male and 20 female adult *Ixodes scapularis* collected in the field from southern Rhode Island, USA, during the fall of 2009. The percentage of ticks infected with *Bb* was 57 %, as determined by direct fluorescent microscopy and using labelled *Bb* antibodies as previously described [27]. The ticks were placed on the back of the dogs and allowed to attach freely. No count of attached ticks was made to reduce the manipulation of animals and ticks, and dogs were further fitted with Elizabethan collars during the tick infestation period to avoid the removal of the ticks through grooming. Ticks were allowed to feed until repletion. At the end of the challenge phase, all still attached ticks were removed and dogs were treated with two applications of a topical acaricide (Frontline®, Merial) on days 10 and 68 of the study.

On day 68 all animals were weighed to determine individual doses for each antimicrobial used. Subsequently, beginning on day 75, dogs allocated to the treatment groups started to receive antimicrobial treatment. The four treatment groups were (*n* = 8 per group): untreated; doxycycline (50 mg Vibramycin®, or 100 mg Vibra-tabs® West-ward Pharmaceuticals) orally once daily at 10 mg/kg for 31 days; amoxicillin (Amoxi-Tabs®, Zoetis) orally three times a day at 20 mg/kg for 31 days; and cefovecin sodium (Convenia®, Zoetis) at 8 mg/kg body weight by subcutaneous injection in two doses, 14 days apart. One dog in the amoxicillin treatment group was dosed incorrectly, receiving 75 mg rather than 150 mg, on four occasions out of a total of 93 treatments for this dog. Review of the antibody and histopathological data suggest that this had no influence on the overall treatment efficacy and as such the animal was included in the overall analysis.

### Examination of clinical signs and sample collection

Dogs were observed daily for general health using standard terms to record any abnormalities. Dog body temperatures were recorded for three weeks following the end of tick infestation (day 11 through day 32). Dogs were, in addition and specifically, observed daily for the presence or absence of lameness.

On days 48 and 49 (pre-treatment), blood samples were collected for the purpose of determining infection status of dogs using SNAP, Quant C6, and Lyme Multiplex assays. Additionally, two skin punch biopsies were collected from each dog in the dorsal cervical region, near the site where the ticks were released onto the animal. Biopsies were taken using a 4 mm punch biopsy under sedation using Dexdomitor® (dexmedetomidine hydrochloride; Zoetis) and Antisedan® (atipamezole hydrochloride; Zoetis) for reversal. The first punch biopsy was placed in Barbour-Stoenner-Kelly II (BSK II) medium for bacterial culture (data not shown) and the second was immediately frozen by placement on dry ice and stored at −20 °C until DNA isolation and PCR were performed as described [28].

Post-treatment blood samples for serum collection were obtained monthly (days 111, 145, 179, 221, 251, 281, 314) and post-treatment skin biopsies and PCR were performed on days 118, 146, 180 and at the end of the study on day 315.

### Serology and histopathology

Serum samples were used to determine antibodies to *Bb* by SNAP® 4Dx® Test; IDEXX, Westbook, ME (SNAP)), a quantitative ELISA (Lyme Quant C6® Test; IDEXX Westbook, ME (Quant C6)), and the Canine Lyme Multiplex assay (Animal Health Diagnostic Center, College of Veterinary Medicine, Cornell University) as previously described [13]. The latter assay was used to evaluate antibodies against *Bb* outer surface protein (Osp) A, C, F, and C_6_ as previously described [13]. Post mortem samples were collected on day 315 from skin near the tick application site and samples from the joint capsule or synovium from left and right shoulder, elbow, carpus, stifle, and tarsus of each dog were collected and placed in 10 % buffered formalin, and submitted to MPI Research (Mattawan, MI) for histological processing. After fixation, the tissues were trimmed, embedded, sectioned, mounted on glass slides and stained with H&E. Tissues were evaluated using light microscopy by a veterinary pathologist blinded as to the assignment of animals to group or treatment. Descriptive observations were entered into a datasheet indicating the presence and severity of each of the lesions in each tissue.

### Data analysis

Data summaries and analyses of antimicrobial efficacy were done using SAS Release 9.2.2 (SAS Institute, Cary, NC). All hypothesis tests, for data after day 75, were conducted at the 0.05 level of significance (two-tailed).

Animals were considered positive for Quant C6 (U/mL) if the value was ≥ 30, positive for Multiplex (median fluorescent intensity (MFI)) OspC if the values were ≥ 1000 and positive for Multiplex OspF if the values were ≥ 1500. Frequency distributions for sero-conversion data as detected by SNAP (positive, negative), Quant C6 and Multiplex OspC and OspF were calculated for each treatment and time point. Sero-conversion data were compared between treatment groups at each time point using Fisher’s Exact test.

Frequency distributions for biopsy PCR were calculated for each treatment and time. Biopsy PCR data were compared between treatment groups at each time point using Fisher’s Exact test. Frequency distributions of the presence/absence of histopathological changes in the joint/tissue samples associated with *Borrelia* infection were calculated for each treatment and joint/tissue. It was determined for each dog whether or not the animal had any joint/tissues with histopathological changes and whether or not the animal had three or more joints/tissues with histopathological changes. Frequency distributions of these variables were calculated for each treatment and compared using Fisher’s Exact test.

## Abbreviations

*Bb*: *Borrelia burgdorferi*
CVB: Center for Veterinary Biologics
IACUC: Institutional Animal Care and Use Committee
LD: Lyme Disease
MIC: Minimum inhibitory concentration
Osp: Outer surface protein
PCR: Polymerase chain reaction
USDA: United States Department of Agriculture

## References

[1] Littman MP, Goldstein RE, Labato MA, Lappin MR, Moore GE (2006). ACVIM small animal consensus statement on Lyme disease in dogs: diagnosis, treatment, and prevention. J Vet Intern Med.

[2] Krupka I, Straubinger RK (2010). Lyme borreliosis in dogs and cats: background, diagnosis, treatment and prevention of infections with Borrelia burgdorferi sensu stricto. Vet Clin North Am Small Anim Pract.

[3] Little SE, Heise SR, Blagburn BL, Callister SM, Mead PS (2010). Lyme borreliosis in dogs and humans in the USA. Trends Parasitol.

[4] Van Vlaenderen I, Nautrup BP, Gasper SM (2011). Estimation of the clinical and economic consequences of non-compliance with antimicrobial treatment of canine skin infections. Prev Vet Med.

[5] Stegemann MR, Sherington J, Blanchflower S (2006). Pharmacokinetics and pharmacodynamics of cefovecin in dogs. J Vet Pharmacol Ther.

[6] Stegemann MR, Passmore CA, Sherington J, Lindeman CJ, Papp G, Weigel DJ (2006). Antimicrobial activity and spectrum of cefovecin, a new extended- spectrum cephalosporin, against pathogens collected from dogs and cats in Europe and North America. Antimicrob Agents Chemother.

[7] Appel MJ, Allan S, Jacobson RH, Lauderdale TL, Chang YF, Shin SJ (1993). Experimental Lyme disease in dogs produces arthritis and persistent infection. J Infect Dis.

[8] Summers BA, Straubinger AF, Jacobson RH, Chang YF, Appel MJ, Straubinger RK (2005). Histopathological studies of experimental lyme disease in the dog. J Comp Pathol.

[9] Chang YF, Novosel V, Chang CF, Summers BA, Ma DP, Chiang YW (2001). Experimental induction of chronic borreliosis in adult dogs exposed to Borrelia burgdorferi-infected ticks and treated with dexamethasone. Am J Vet Res.

[10] Straubinger RK, Straubinger AF, Summers BA, Jacobson RH, Erb HN (1998). Clinical manifestations, pathogenesis, and effect of antibiotic treatment on Lyme borreliosis in dogs. Wien Klin Wochenschr.

[11] Straubinger RK, Straubinger AF, Summers BA, Jacobson RH (2000). Status of Borrelia burgdorferi infection after antibiotic treatment and the effects of corticosteroids: An experimental study. J Infect Dis.

[12] Susta L, Uhl EW, Grosenbaugh DA, Krimer PM (2012). Synovial lesions in experimental canine Lyme borreliosis. Vet Pathol.

[13] Wagner B, Freer H, Rollins A, Garcia-Tapia D, Erb HN, Earnhart C (2012). Antibodies to Borrelia burgdorferi OspA, OspC, OspF, and C6 antigens as markers for early and late infection in dogs. Clin Vaccine Immunol.

[14] Levy SA, O’Connor TP, Hanscom JL, Shields P, Lorentzen L, Dimarco AA (2008). Quantitative measurement of C6 antibody following antibiotic treatment of Borrelia burgdorferi antibody-positive nonclinical dogs. Clin Vaccine Immunol.

[15] Levy S, O’Connor TP, Hanscom JL, Shields P (2002). Utility of an in-office C6 ELISA test kit for determination of infection status of dogs naturally exposed to Borrelia burgdorferi. Vet Ther.

[16] Littman MP (2013). Lyme nephritis. J Vet Emerg Crit Care (San Antonio).

[17] Gerber B, Haug K, Eichenberger S, Reusch CE, Wittenbrink MM (2009). Follow-up of Bernese Mountain dogs and other dogs with serologically diagnosed Borrelia burgdorferi infection: what happens to seropositive animals?. BMC Vet Res.

[18] Gerber B, Eichenberger S, Haug K, Wittenbrink MM, Reusch CE (2009). Association of urine protein excretion and infection with Borrelia burgdorferi sensu lato in Bernese Mountain dogs. Vet J.

[19] Horney BS, Stojanovic V (2013). Protein-losing nephropathy associated with Borrelia burgdorferi seropositivity in a soft-coated wheaten terrier: response to therapy. Can Vet J.

[20] Goldstein RE, Brovida C, Fernandez-Del Palacio MJ, Littman MP, Polzin DJ, Zatelli A (2013). Consensus recommendations for treatment for dogs with serology positive glomerular disease. J Vet Intern Med.

[21] Straubinger RK, Summers BA, Chang YF, Appel MJ (1997). Persistence of Borrelia burgdorferi in experimentally infected dogs after antibiotic treatment. J Clin Microbiol.

[22] Dever LL, Jorgensen JH, Barbour AG (1992). In vitro antimicrobial susceptibility testing of Borrelia burgdorferi: a microdilution MIC method and time-kill studies. J Clin Microbiol.

[23] Levin JM, Nelson JA, Segreti J, Harrison B, Benson CA, Strle F (1993). In vitro susceptibility of Borrelia burgdorferi to 11 antimicrobial agents. Antimicrob Agents Chemother.

[24] Ruzic-Sabljic E, Podreka T, Maraspin V, Strle F (2005). Susceptibility of Borrelia afzelii strains to antimicrobial agents. Int J Antimicrob Agents.

[25] Sicklinger M, Wienecke R, Neubert U (2003). In vitro susceptibility testing of four antibiotics against Borrelia burgdorferi: a comparison of results for the three genospecies Borrelia afzelii, Borrelia garinii, and Borrelia burgdorferi sensu stricto. J Clin Microbiol.

[26] Brissette CA, Kees ED, Burke MM, Gaultney RA, Floden AM, Watt JA (2013). The multifaceted responses of primary human astrocytes and brain microvascular endothelial cells to the Lyme disease spirochete, Borrelia burgdorferi. ASN Neuro.

[27] Nicholson MC, Mather TN, Donnelly EF (1996). Lyme disease in Rhode Island: three years of surveillance. Med Health Rhode Island.

[28] Rhodes DV, Earnhart CG, Mather TN, Meeus PF, Marconi RT (2013). Identification of Borrelia burgdorferi ospC genotypes in canine tissue following tick infestation: implications for Lyme disease vaccine and diagnostic assay design. Vet J.

